# Enhanced feature selection and ensemble learning for cardiovascular disease prediction: hybrid GOL2-2 T and adaptive boosted decision fusion with babysitting refinement

**DOI:** 10.3389/fmed.2024.1407376

**Published:** 2024-07-05

**Authors:** S. Phani Praveen, Mohammad Kamrul Hasan, Siti Norul Huda Sheikh Abdullah, Uddagiri Sirisha, N. S. Koti Mani Kumar Tirumanadham, Shayla Islam, Fatima Rayan Awad Ahmed, Thowiba E. Ahmed, Ayman Afrin Noboni, Gabriel Avelino Sampedro, Chan Yeob Yeun, Taher M. Ghazal

**Affiliations:** ^**1**^Department of Computer Science and Engineering, Prasad V Potluri Siddhartha Institute of Technology, Vijayawada, India; ^2^Faculty of Information Science and Technology, University Kebangsaan Malaysia, Bangi, Selangor, Malaysia; ^3^Department of Computer Science and Engineering, Sir C R Reddy College of Engineering, Eluru, India; ^4^Institute of Computer Science and Digital innovation, UCSI University, Kuala Lumpur, Malaysia; ^5^Computer Science Department, College of Computer Engineering and Science, Prince Sattam Bin Abdulaziz University, Al-Kharj, Saudi Arabia; ^6^Computer Science Department, College of Science and Humanities-Jubail, Imam Abdulrahman Bin Faisal University, Dammam, Saudi Arabia; ^7^Department of Surgery, Medical College For Women and Hospital, Dhaka, Bangladesh; ^8^Faculty of Information and Communication Studies, University of the Philippines Open University, Los Baños, Philippines; ^9^Center for Computational Imaging and Visual Innovations, De La Salle University, Manila, Philippines; ^10^Centre for Cyber Physical Systems, Computer Science Department, Khalifa University, Abu Dhabi, United Arab Emirates

**Keywords:** multivariate imputation by chained equations, synthetic minority over-sampling technique, interquartile range, adaptive boosted decision fusion, cardiovascular disease, adaboost decision fusion (ABDF)

## Abstract

**Introduction:**

Global Cardiovascular disease (CVD) is still one of the leading causes of death and requires the enhancement of diagnostic methods for the effective detection of early signs and prediction of the disease outcomes. The current diagnostic tools are cumbersome and imprecise especially with complex diseases, thus emphasizing the incorporation of new machine learning applications in differential diagnosis.

**Methods:**

This paper presents a new machine learning approach that uses MICE for mitigating missing data, the IQR for handling outliers and SMOTE to address first imbalance distance. Additionally, to select optimal features, we introduce the Hybrid 2-Tier Grasshopper Optimization with L2 regularization methodology which we call GOL2-2T. One of the promising methods to improve the predictive modelling is an Adaboost decision fusion (ABDF) ensemble learning algorithm with babysitting technique implemented for the hyperparameters tuning. The accuracy, recall, and AUC score will be considered as the measures for assessing the model.

**Results:**

On the results, our heart disease prediction model yielded an accuracy of 83.0%, and a balanced F1 score of 84.0%. The integration of SMOTE, IQR outlier detection, MICE, and GOL2-2T feature selection enhances robustness while improving the predictive performance. ABDF removed the impurities in the model and elaborated its effectiveness, which proved to be high on predicting the heart disease.

**Discussion:**

These findings demonstrate the effectiveness of additional machine learning methodologies in medical diagnostics, including early recognition improvements and trustworthy tools for clinicians. But yes, the model’s use and extent of work depends on the dataset used for it really. Further work is needed to replicate the model across different datasets and samples: as for most models, it will be important to see if the results are generalizable to populations that are not representative of the patient population that was used for the current study.

## 1 Introduction

Many communities are affected by heart disease, a major global health problem that is responsible for many cases of sickness and death. There is an increasing need to understand the complexity of heart diseases as our understanding of cardiovascular health expands. Think about this: someone dies of cardiovascular issues every 37 s in America, which highlights the urgency to quell this unseen epidemic (American Heart Association, 2022). This mind-boggling figure shows how huge numbers of people, families, and societies are affected by cardiac diseases (1).

The human heart is one fantastic example of biologically engineered machinery that coordinates life’s intricate workings by driving vital energy through a network of complex vessels. However, repercussions can be disastrous when this symphony gets disrupted. Heart problems include conditions like coronary artery disease, heart failure, arrhythmias and congenital malformations. Their etiology is multifactorial involving genetic predispositions, behavioral factors and countless sophisticated biochemical pathways (2). Beyond the confines of medical practice, heart diseases contain a rich assortment of stories—chronicles of courage, sadness and hope. Every heartbeat affects those whose lives are touched by it and every diagnosis carries along its own path for each of them which are distinct and personal.

A major global health issue, cardiovascular disease, and cardiovascular disorders. Coronary artery disease (CAD), the most common, causes narrowing or blockage of the coronary arteries, leading to angina or myocardial infarction. Heart failure reduces oxygen delivery because the heart cannot pump blood properly. Mild exercise causes an abnormal heart rate that can impair circulation. Valvular heart disease damages the heart muscles and limits blood flow. Cardiomyopathy occurs when the heart muscle contracts or stiffens, reducing its ability to carry blood (3).

Poor diet, lack of physical activity, tobacco use, alcohol abuse and obesity are major risk factors. Heart disease prevention includes healthy eating, exercise, weight control, and smoking cessation. Treatment options range from medical to surgical, depending on the severity. Routine inspections detect and address them quickly (4). Knowing the risk factors and prioritizing cardiovascular health helps reduce the impact of cardiovascular disease.

Risk factors for cardiovascular disease include smoking and alcohol misuse. Coronary artery disease (5), hypertension, decreased oxygen saturation, and accelerated blood clotting are all consequences of smoking. Consuming alcohol raises the risk of hypertension, heart disease (6, 7), and cholesterol. When smoked and drunk at the same time, oxidative stress rises, the immune system is weakened, and blood arteries and cholesterol are damaged. Heart disease, particularly myocardial, cerebral, and cardiac insufficiency, is greatly increased by this lethal combination. It is vital to quit smoking, restrict alcohol intake, and maintain cardiovascular health since these habits add up to a lot of harm. Although beating addiction could be difficult, the rewards in terms of heart health are substantial.

Adaptive enhanced decision fusion is crucial for disease prediction, especially in cardiovascular health. Combining numerous models and adjusting to changing data patterns enhances early disease detection and prediction. The ABDF educates doctors on cardiac illnesses to help them choose the best treatments and improve patient outcomes. In the complex realm of cardiovascular diseases, its versatility allows quick risk assessment and appropriate intervention. ABDF is a cutting-edge ensemble learning approach that enhances cardiovascular health patient care and predictive analytics.

**Figure 1:**
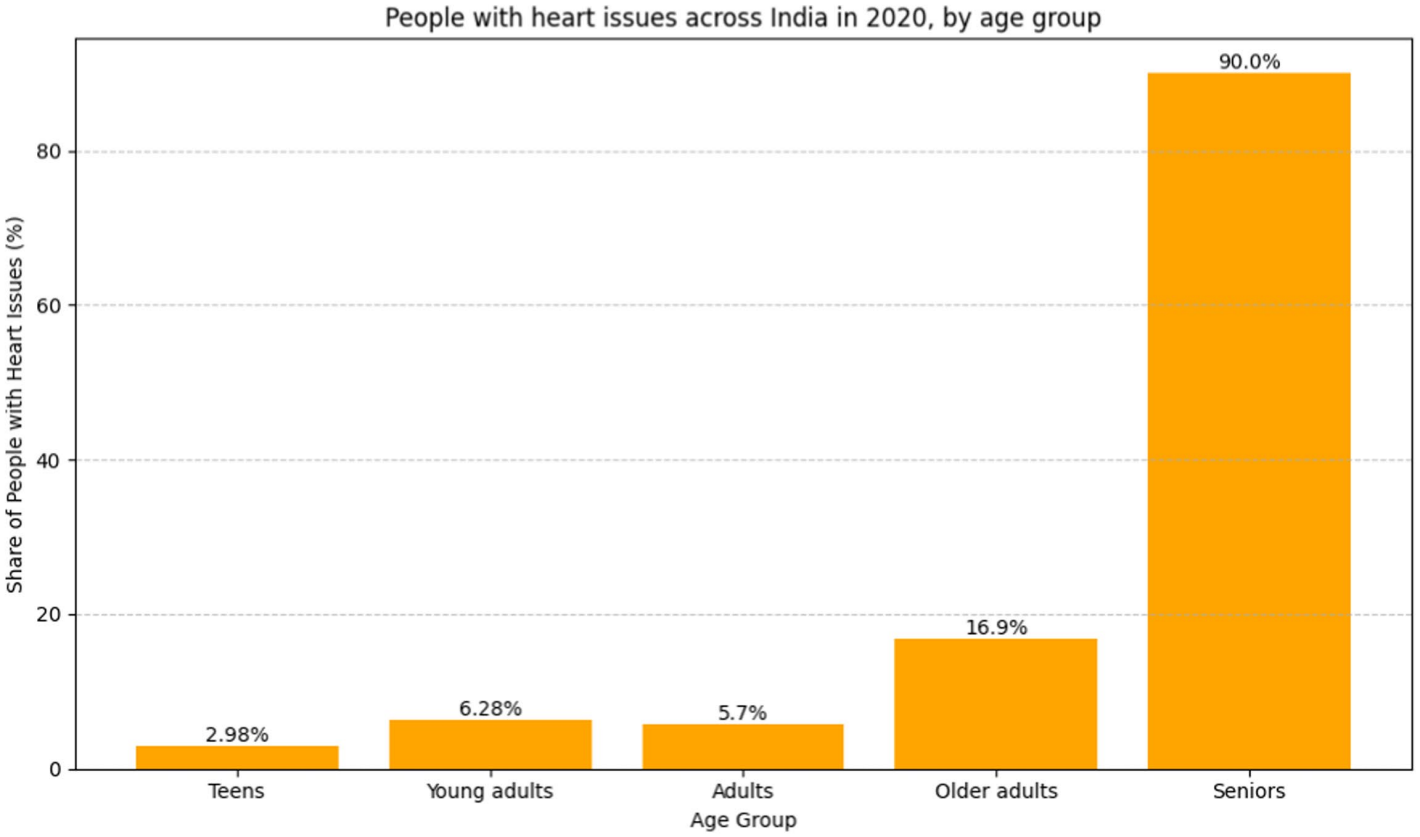
Bar graph people with heart issues across India in 2020, by age group (8) (https://www.statista.com).

As data reveals, the cardiovascular problem percentage among people in India as diagnosed in the year 2020 is shown in Figure 1, using the breakdown by age group. In cardiovascular matters, most often, the older age group was seen having more frequent problems than the younger age group. The rate of cardiovascular disease found among the teenagers of the age group below 19 is about 2.98%, which is comparably lower compared to that of the young people of the age group 20–29, which registers about 5%. Investigators have been able to ascertain that the 45- to 59-year-old population group had an illness rate of cardiovascular problems of about 11.9%, while that of the 30- to 44-year-old group was about 6.28%. At a rate of 18.7%, the above-60-year-old succession group accounts for the highest prevalence of cardiovascular diseases. Given the existence of age disparities, policymakers should focus on the development of auxiliary policies, early detection, effective healthcare delivery, and educational campaigns that will help in the ongoing battle against the rising frequency of cardiovascular diseases among the aging population (8–12).

## 2 Literature review

In 2020, Shah et al. (13) examine data mining and machine learning for heart disease prediction. The study stresses the need of precise and timely identification of heart disease, a top worldwide mortality. Using the enormous Cleveland database of UCI repository, 303 cases and 76 characteristics are rigorously condensed to 14 important elements. The study compares popular algorithms including Naïve Bayes, decision tree, K-nearest neighbor (KNN), and random forest for heart disease prediction. KNN was the most accurate algorithm, demonstrating predictive modeling potential. The finding agrees with earlier research that many algorithms are needed for complete findings. Future data mining approaches such time series analysis, clustering, association rules, support vector machines, and evolutionary algorithms are suggested to improve predicted accuracy. While insightful, the paper admits its limits and advocates for further research to improve early and accurate heart disease prediction algorithms.

In 2020, Katarya et al. (14) conducted a survey saying that heart disease is a global issue with rising treatment expenses, therefore early detection is essential. Alcohol, tobacco, and inactivity are essential heart disease indicators. The paper recommends using machine learning, particularly supervised methods, for healthcare decision-making and prediction to address this essential issue. Several algorithms, including as ANN, DT, RF, SVM, NB, and KNN, being investigated for heart disease prediction. The research summarizes these algorithms’ performance to reveal their efficacy. In conclusion, automated technologies to anticipate cardiac disease early on help healthcare professionals diagnose and empower patients to monitor their health. Feature selection is critical, and hybrid grid search and random search are suggested for optimization. Search algorithms for feature selection and machine learning will improve cardiac disease prediction, leading to better healthcare treatments, according to the report.

In 2021, Jindal et al. (15) highlights the increasing number of heart diseases and the need for prediction models. The declaration acknowledges the challenge of correct diagnosis and promotes machine learning techniques for accurate projections. Logistic regression and KNN are compared to naive Bayes in the research. The proposed heart disease prediction system reduces costs and improves medical care. The research also includes a Logistic Regression, Random Forest Classifier, and KNN cardiovascular disease detection model. The model’s accuracy is 87.5%, up from 85% for previous models. The literature shows that the KNN method outperforms other algorithms with an accuracy rate of 88.52%. The article claims that machine learning can predict cardiac issues more accurately than conventional techniques, improving patient care and lowering costs.

In 2019, Gonsalves et al. (16) uses Machine Learning (ML) approaches such as Naïve Bayes (NB), Support Vector Machine (SVM), and Decision Tree (DT) to predict Coronary Heart Disease (CHD). Coronary heart disease (CHD) is a major cause of death around the world, highlighting the need of early detection. The work uses historical medical data and three supervised learning approaches to discover CHD data correlations to improve prediction precision. The summary of the literature acknowledges the complexity of medical data and CHD prediction linkages, stressing the challenges of existing techniques. The study’s focus on NB, SVM, and DT matches existing research techniques, highlighting the availability of disease prediction machine learning algorithms. Early screening and identification are crucial for patient well-being, resource allocation, and preventative interventions, according to the research. The discussion of ML model performance, including accuracy, sensitivity, specificity, and other characteristics, sheds light on Naive Bayes, Support Vector Machines, and Decision Trees. Despite not meeting threshold rates, the Naive Bayes (NB) classifier looks to be the best option for the dataset. According to the literature review, unsupervised learning and data imbalance should be studied in the future. This will enhance prediction algorithms and may lead to mobile CHD diagnosis apps.

In 2018, Nashif et al. (17), addresses cardiovascular problems across the globe and highlights the necessity to detect and monitor them early. The cloud-based heart disease prediction system uses powerful machine learning. Interestingly, the Support Vector Machine (SVM) method has 97.53% accuracy. Real-time patient monitoring using Arduino for data collection is presented in the study, focusing on remote healthcare. Comparative evaluations show SVM outperforms other models. The abstract concludes with potential issues including photoplethysmography-based blood pressure monitoring. The literature analysis highlights cloud-based prediction and real-time patient monitoring as a solution to PPG-based system constraints.

In 2023, Bhatt et al. (18) used Machine learning to create a cardiovascular disease prediction model. The study employed 70,000 Kaggle-downloaded real-world samples. Huang initialization improves k-modes clustering classification accuracy. GridSearchCV optimizes random forest, decision tree, multilayer perceptron, and XGBoost models. With 86.37 to 87.28% accuracy, the models are great. Multiple layer perceptron outperforms other models. The study adjusts age, blood pressure, and gender to account for heart disease progression. Despite promising outcomes, the study had limitations. These include employing a single dataset, only considering particular clinical and demographic features, and not comparing results to other test datasets. More research is needed to overcome these restrictions, compare clustering algorithms, test the model on new data, and improves interpretability. Machine learning—particularly clustering algorithms—can effectively predict cardiac illness and guide focused treatment and diagnostic measures.

In 2023, Abood Kadhim et al. (19) examines the growing use of artificial intelligence—specifically machine learning—in cardiac disease diagnosis and prediction. Support vector machines, random forests, and logistic regression are tested on Cleveland Clinic data. Research on artificial intelligence in cardiac care is also examined. The study found that support vector machines are the most accurate heart disease diagnosis tools at 96%. It also presents a 95.4% accurate random forest model for cardiac attacks. The findings demonstrate the importance of AI in healthcare decision-making and early cardiac problem intervention.

**Figure 2:**
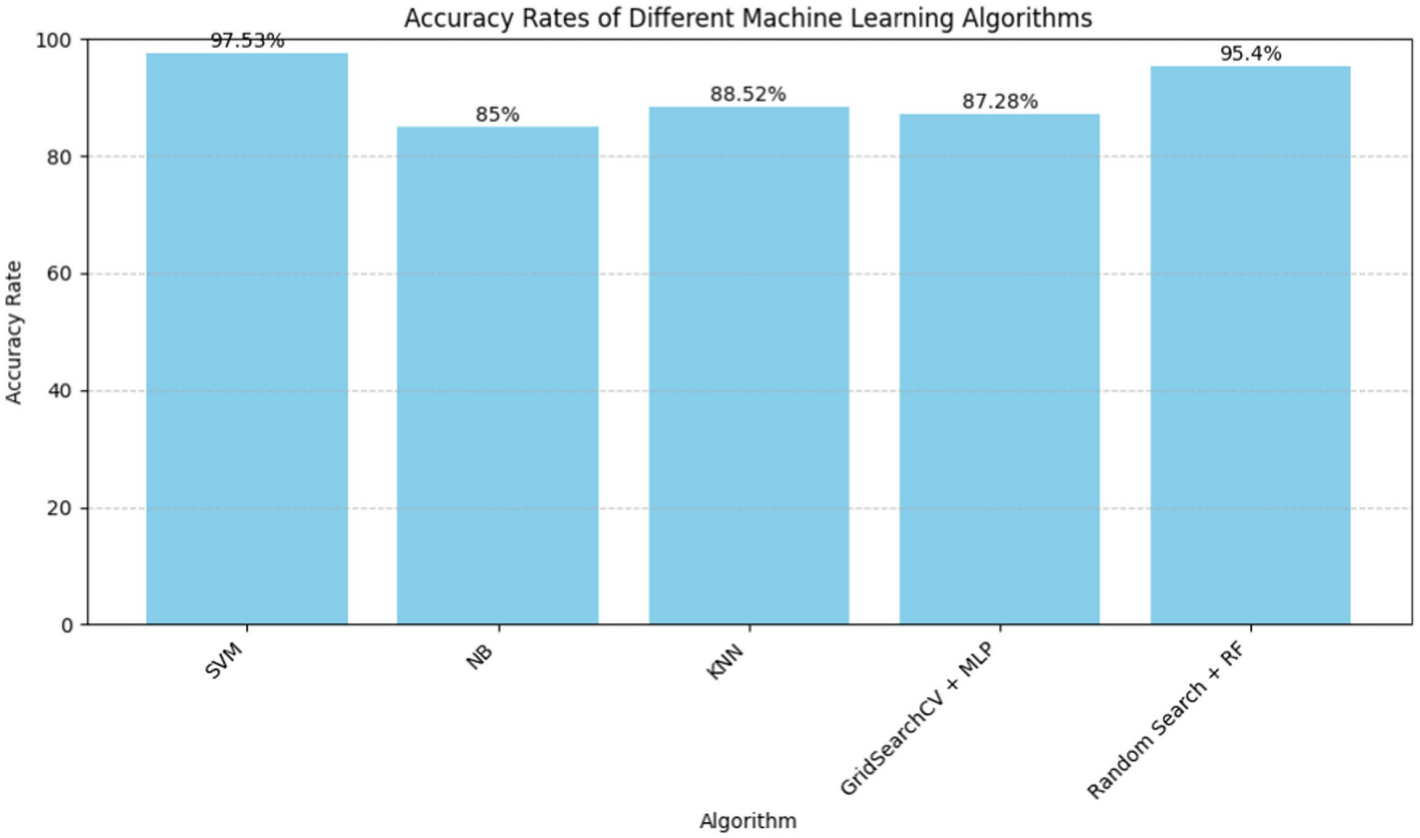
Machine learning algorithms for heart disease prediction.

Recent researches have stressed the need for global cardiovascular disease diagnosis and identification. Several papers in 2020 and 2021 studied Naïve Bayes, decision tree, K-nearest neighbor (KNN), and random forest algorithms using data mining and machine learning methods. The primary findings are that K-Nearest Neighbors (KNN) may predict heart disease, that supervised machine learning may make healthcare decisions, and that logistic regression, KNN, and naive Bayes are comparable. These findings show the usefulness of predictive models in addressing the rising number of cardiac ailments, leading to healthcare technology advances for early identification and better patient treatment (Figure 2).

### 2.1 Motivation

Due to the global the amount of cardiovascular diseases, data mining and machine learnnng research on heart disease prediction is escalating. Heart disease is the most common cause of mortality worldwide. To reduce mortality rates, these medical conditions must be accurately and quickly detected. Researchers are studying machine learning to improve diagnostic skills since conventional methods frequently make inaccurate predictions. These studies aim to enhance early diagnosis and treatment. Medical data is complex and risk variables change, making machine learning an intriguing method for finding meaningful patterns and improving heart disease prediction.

### 2.2 Research gap

Despite the wealth of knowledge in machine learning approaches to heart disease prediction, additional research is needed. Shah et al. (13), Katarya et al. (14), Jindal et al. (15), Gonsalves et al. (16), Nashif et al. (17), Bhatt et al. (18), and Abood Kadhim et al. (19) all emphasize the importance of accurate and early heart disease detection. These researches have examined how K-nearest neighbor (KNN), Support Vector Machine (SVM), Random Forest, and logistic regression can increase predicted accuracy. These attempts are intriguing, but they also highlight limits like dataset dependence, feature selection optimization issues, and the need for more unsupervised learning research. Address data imbalance and real-time patient monitoring equipment concerns. Thus, even though machine learning could change cardiac illness prediction, more research is needed to improve algorithms, overcome data constraints, and improve cardiovascular health care outcomes. The current study lacks detailed algorithm assessments, leaving the best technique for exact predictions unknown. There is also insufficient research into using advanced data mining methods like time series analysis and evolutionary algorithms to better forecast heart illness. Overcome these gaps to increase prediction model robustness and precision in this critical healthcare sector.

The research’s scope is to create trustworthy and effective cardiovascular disease diagnostic tools. Our goal is to reduce heart disease deaths and improve heart disease predictions using powerful machine learning.

- SMOTE, IQR outlier identification, and MICE are used to solve data difficulties in this work. We also introduce Hybrid GOL2-2 T, a hybrid feature selection approach.
- It uses L2 regularization and the Grasshopper Optimization Algorithm.
- A babysitter algorithm and Adaptive Boosted Decision Fusion (ABDF) ensemble learning increase predictive modeling accuracy.
- Our model will be assessed by accuracy, recall, and AUC score.

The main goal of this project is to develop reliable diagnostic tools for early diagnosis and treatment of cardiovascular diseases. This can help doctors improve patient outcomes and reduce illness.

In the subsequent sections, Section 2 provides a comprehensive literature analysis of the corpus of recent publications. The suggested methodology is then presented in Section 3. Section 4 offers a thorough summary of the results and the discussion that follows. In Section 5, prospective avenues for further research are explored and the article is summarized with a conclusion.

## 3 Proposed methodology

For the two-tier Feature Selection Hybrid GOL2-2 T, starting from the data pre-processing stage among the partitions, 70% of the data partition is allotted for the training set and 30% for the testing set. An objective under this category makes it easy to evaluate the performance of the models in question based on it deeply. The second to the last step is the missing data estimate, which makes use of the Multivariate Imputation by Chained Equations (MICE) approach. This, in return, ensures the completeness of information from one or many variables. In this case, the following techniques were corrected with a deficiency of training the model and have high interoperability with the techniques of machine learning; Imputation, Data scaling, and Label encoding. Inside the method, it has the Inter Quartile Range (IQR) to identify and deal with an outlier in an effort to enhance the resilience of the model through a reduction in influence that emanates from abnormal data points. The major maxim is SMOTE, which a synthetic minority is over-sampling technique aimed at the problem of class imbalance. The technique established a fair representation through the development of synthetic minorities, toward the reduction of biases that may associate with the general over-representation of the dominant class.

**Figure 3:**
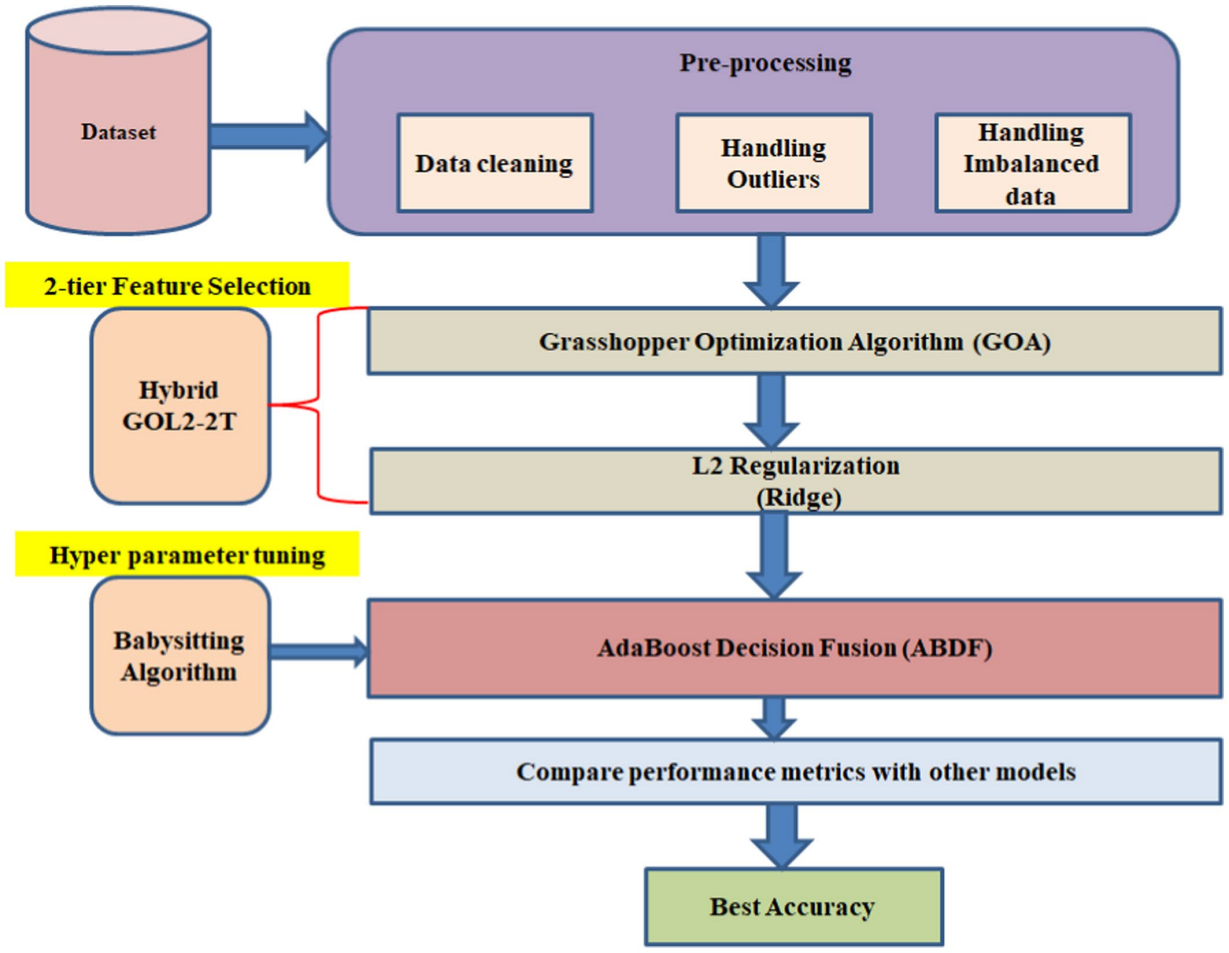
Heart disease forecasting workflow.

2-tier Feature Selection is based on the L2 Regularization (Ridge) (20) along with the Grasshopper Optimization (GOA) method; therefore, the proposed Hybrid GOL2-2 T model is going to form a 2-level model for Feature Selection. It also employs ABDF hyperparameters, which have been babysitting algorithm to be fine-tuned after proper pre-processing of the dataset. Therefore, AdaBoost Decision Fusion (ABDF) maximizes the predictive modeling tasks’ accuracies by pulling the performance measures out with respect to other models for comparison (Figure 3).

### 3.1 Data collection

**Figure 4:**
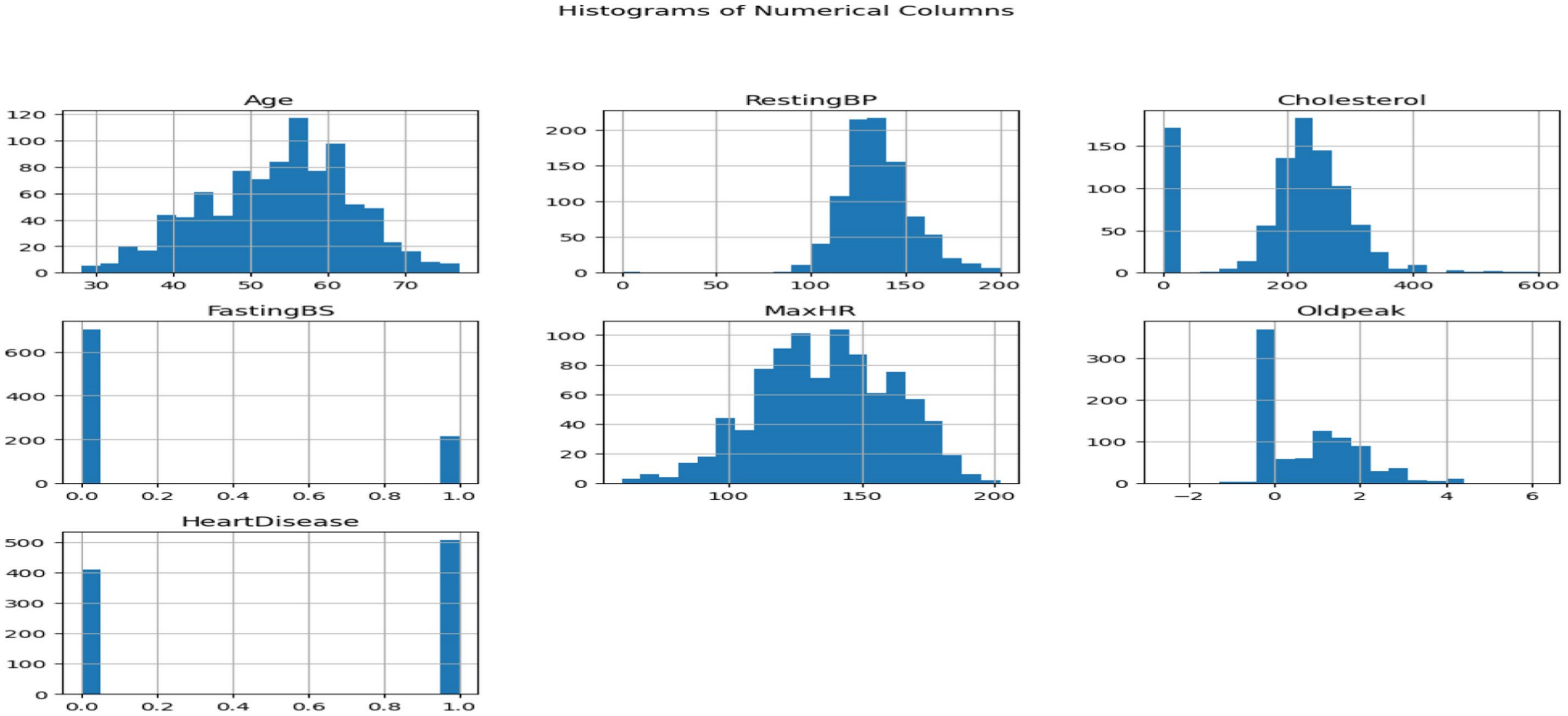
Histograms of numeric columns.

The 1988 heart disease dataset (21) is an excellent resource for studying and forecasting cardiovascular disease prevalence. Age, gender, type of chest pain, blood pressure, cholesterol levels, and the presence of numerous cardiovascular diseases are among the 14 important factors. There is a large variety of ages represented in the dataset, with the majority falling between 40 and 60. Of those, 207 are male and 96 are female. With a value of 1 for males and 0 for females, the variable “sex” is included in the data for each issue as an essential health indicator. While we display resting blood pressure (trestbps) and serum cholesterol levels (chol) as whole numbers, we categorize chest discomfort as 1, 2, 3, or 0. Exang, exercise-induced angina, exercise-induced ST depression compared to rest, the slope of the peak exercise ST segment, the number of major vessels colored by fluoroscopy, and thalassemia type are some other factors that improve the dataset. In order to promote a thorough study of cardiovascular health and facilitate the development of reliable prediction systems, the “target” property shows whether heart disease is present (1) or absent (0) (Figure 4).

#### 3.1.1 Visualizing the attributes of heart disease dataset using pair plot

This dataset encompasses six numerical variables: RestingBP, Cholesterol, FastingBS, MaxHR, Oldpeak. Two variables are distributed in each grid subplot. Variable correlations in the Heart Disease dataset are shown in the pair plot. The correlation between two variables is displayed in every matrix scatterplot. The level of heart disease dictates the color of the dots. Early detection of data patterns and trends can be aided by this. It can reveal whether there are commonalities between those who have cardiac disease and those who do not.

**Figure 5:**
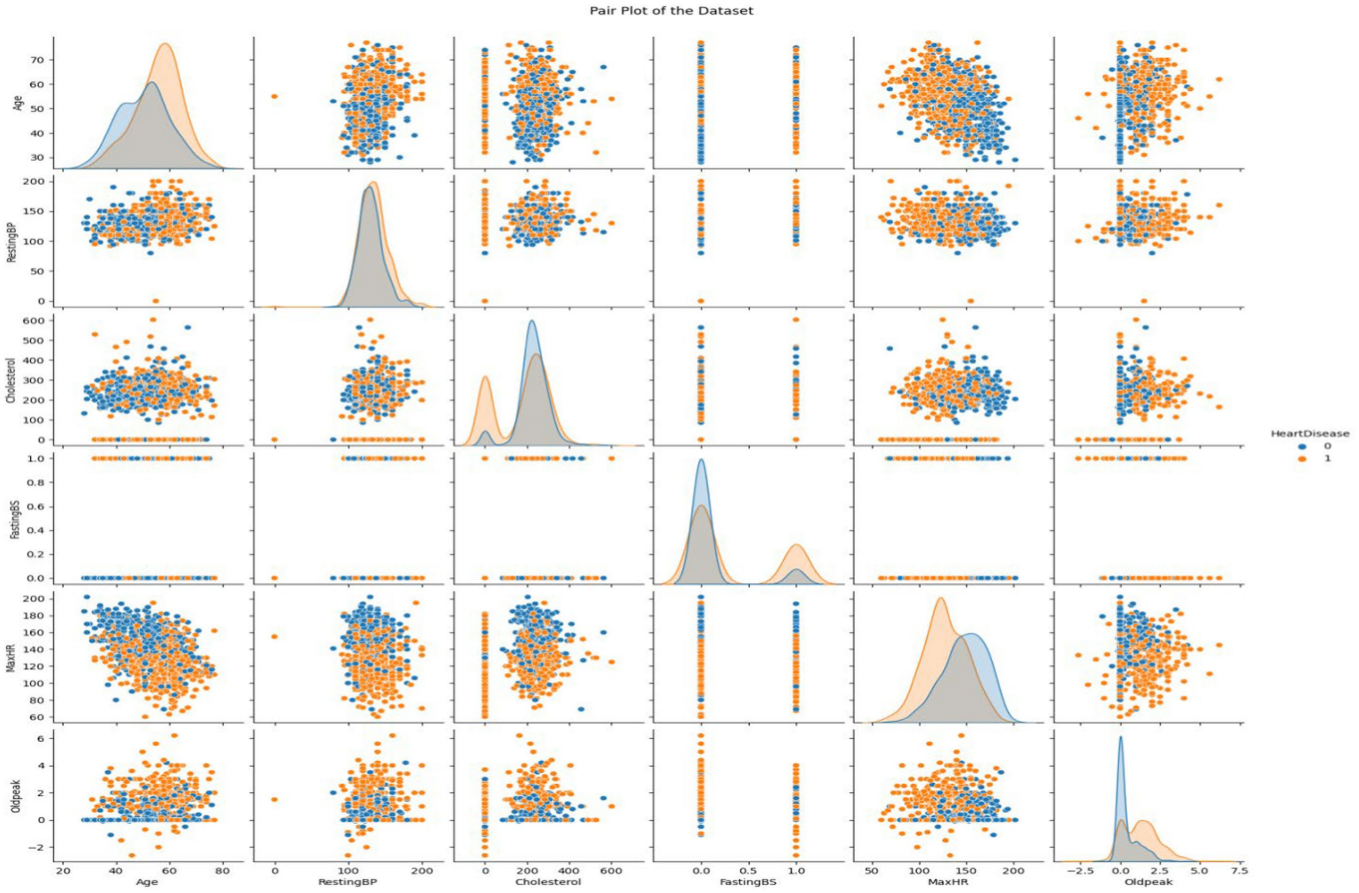
Visualizing the attributes of heart disease dataset using pair plot.

Histograms show variable distribution, while scatter plots show the connection between paired variables. In the upper left subplot, RestingBP distribution is presented. The y-axis shows data point frequency, and the x-axis shows RestingBP levels. The bottom right subplot displays the association between MaxHR and Oldpeak, an off-diagonal plot. This subplot shows Oldpeak on the *y*-axis and MaxHR on the x-axis. Examining the pair plot can reveal patterns and linkages, such as cholesterol-resting blood pressure correlations. This graphical tool simplifies dataset analysis, especially for outliers and linear correlations. We consider non-diagonal scatter plots while examining linear relationships. Straight lines between scatter plot dots indicate the variables’ direction and strength. Outliers are scatter plot data points far from the main cluster. If we want to use machine learning to forecast cardiac disease from patient data, we need to understand these tendencies. It might be necessary to make adjustments and do further research on visual representations in order to have a better understanding of the dataset (Figure 5).

### 3.2 Pre-processing

#### 3.2.1 Data cleaning with MICE

Data pretreatment requirements include cleaning the data to ensure dataset correctness and completeness and that it is analysis or model training ready. Absent data often hurts machine learning models. MICE (22) handle missing data thoroughly and statistically through Multiple Imputation by Chained Equations shown in equation (1). In an iterative process, MICE calculate conditional distributions for all variables with missing data using observed data and other variable imputations. As iterations continue until convergence, the process creates various entire datasets. To accommodate for missing value uncertainty, each dataset has its own imputations. Multiple Imputation by Chained Equations (MICE) works well for non-random missing data patterns in real-world datasets where observed values may affect missing. It evaluates variables and predicts data distributions. The MICE technique provides imputations, updates models, and combines findings to provide credible imputed datasets. Finalized datasets can be used to train machine-learning models. MICE address missing data to improve model performance and assure unbiased parameter estimates.


(1) $$y\wedge_{ji}^{imputed}=f\left(x_{i,-j}\right)+\in_{i}\;$$

-
$y\wedge_{ji}^{imputed}\;$ shows the value that has been ascribed to the absent item.
- *f*: The missing value is estimated by the function. The data type of variable j might affect this function.
-
$x_{i,-j}$ : With the exception of variable j, all observed values of the variables are represented by the vector in the *i*th observation.
-
$\in_{i}:$ Error term

The observed values of all the variables in this context, with the exception of variable *j* in observation *i*, are stored in the vector $\;x_{i,-j}$ . By using these observed values, the function *f* is used to estimate the missing value. The assumed value’s error word $\in_{i}\;$ denotes any inexplicable volatility or unpredictability.

#### 3.2.2 Scaling with label encoder

There are two essential methods for preparing machine learning data: label encoding and scaling. To transform categorical data into a numerical form, Label Encoding assigns unique integer labels to each category. One method for giving numerical values to categorical variables is Label Encoding (23). With Label Encoding, “Male” and “Female” would be represented as 0 and 1, respectively, in a “Gender” column. For algorithms that can only take numerical input, this simplifies the usage of categorical variables. On the flip side, numerical features can be scaled to be uniform in size so that no one characteristic can have an outsized impact due to size disparities. Model convergence and performance are both enhanced by methods Standard Scaling, [shown in equation (2)] which ensure that all features contribute equally. A typical preprocessing step involves converting categorical characteristics using Label Encoding and then scaling numerical features to make their magnitudes consistent. Label Encoding and Scaling, when used together; make it easy to get datasets ready to be used in machine learning algorithms.

(2) $$X_{scaled}=\frac{X-\mu }{\sigma }\;$$

- The initial feature value was *X*.
- The feature values mean is represented by μ.
- The feature values’ standard deviation is represented by σ.

#### 3.2.3 Handling outliers with IQR

**Figure 6:**
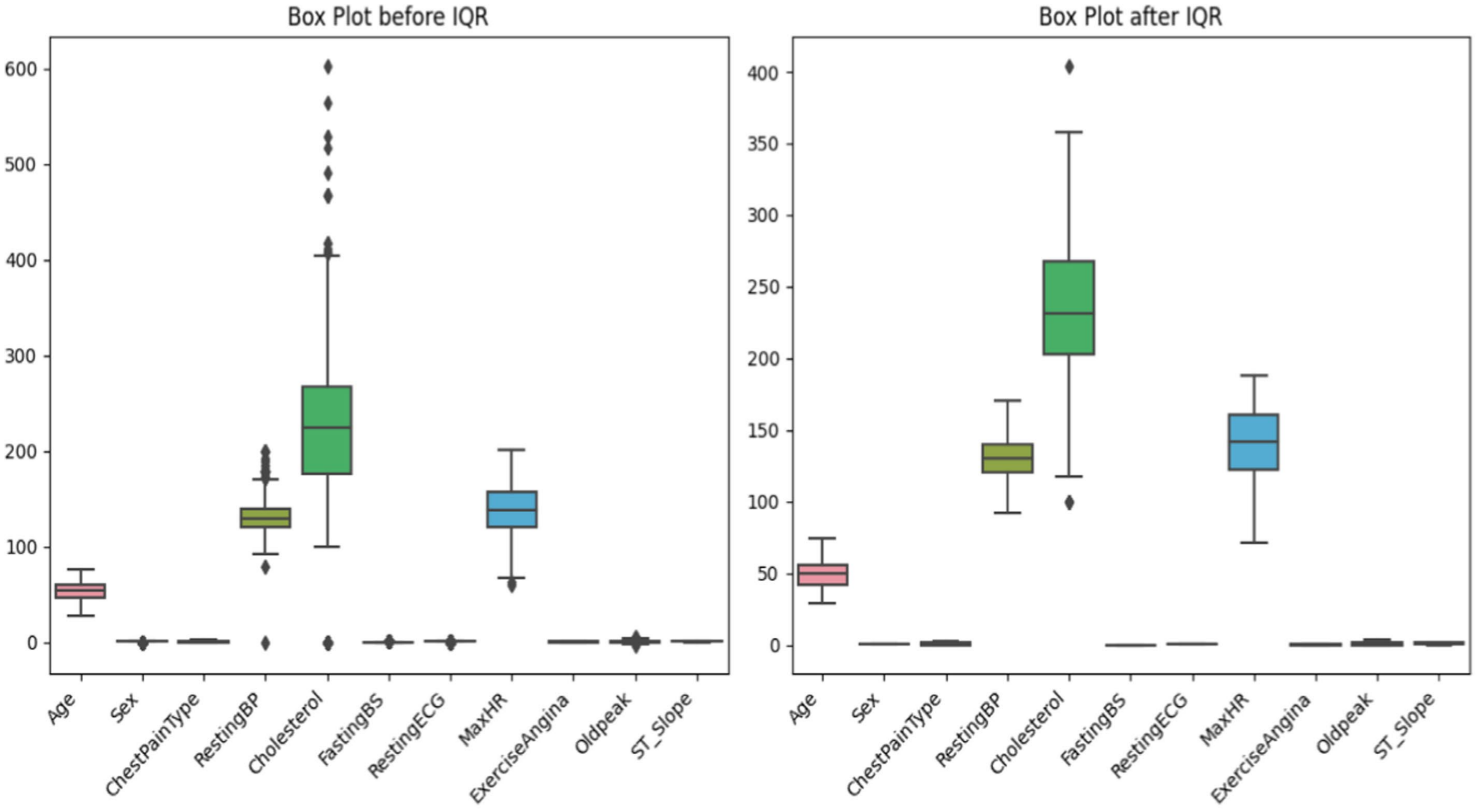
Before and after outlier capping by using IQR.

Careful data preparation, including outlier removal, improves machine learning model durability. Interquartile Range (IQR) is a prominent method for finding and treating dataset outliers. Interquartile range (IQR) is the difference between a distribution’s third and first quartiles, or 75th and 25th percentiles [shown in equation (3)]. Abnormal data points fall below or above the lower and higher limits (Q1–1.5 * IQR and Q3 + 1.5 * IQR, respectively) [shown in equations (4, 5)]. Outliers might hurt the model’s performance, but the IQR-based technique would find and fix them. To minimize outliers’ impact on learning, alter them. This reduces model sensitivity to unexpected data sets. This is crucial for algorithms that respond fast to data distribution changes (Table 1).
The initial stage in IQR-based outlier treatment is splitting the sample into quartiles and determining the IQR (24). Outliers can be deleted or altered by comparing them against boundaries. This technique emphasizes creating more extensive and reliable datasets to improve ML model generalizability and prediction accuracy. The IQR outlier control approach must be used to prepare data for future machine learning experiments to ensure reliability and efficiency (Figure 6).

(3) $${\mathrm{IQR}}_{\mathrm{outlier}}=Q_{3}-Q_{1}$$

(4) $$\mathrm{LowerBound}=Q_{1}-1.5*\mathrm{IQR}\;$$

(5) $$\mathrm{UpperBound}=Q_{3}+1.5*\mathrm{IQR}\;$$

**Table 1:** Machine learning algorithms for heart disease prediction.

Algorithm	Accuracy rate	Citation
SVM	97.53	Nashif et al. (17)
NB	85	Gonsalves et al. (16)
KNN	88.52	Jindal et al. (15)
KNN	90.78	Shah et al. (13)
GridSearchCV + MLP	87.28	Bhatt et al. (18)
Random Search + RF	95.4	Abood Kadhim et al. (19)

#### 3.2.4 Handling imbalanced dataset with SMOTE

**Figure 7:**
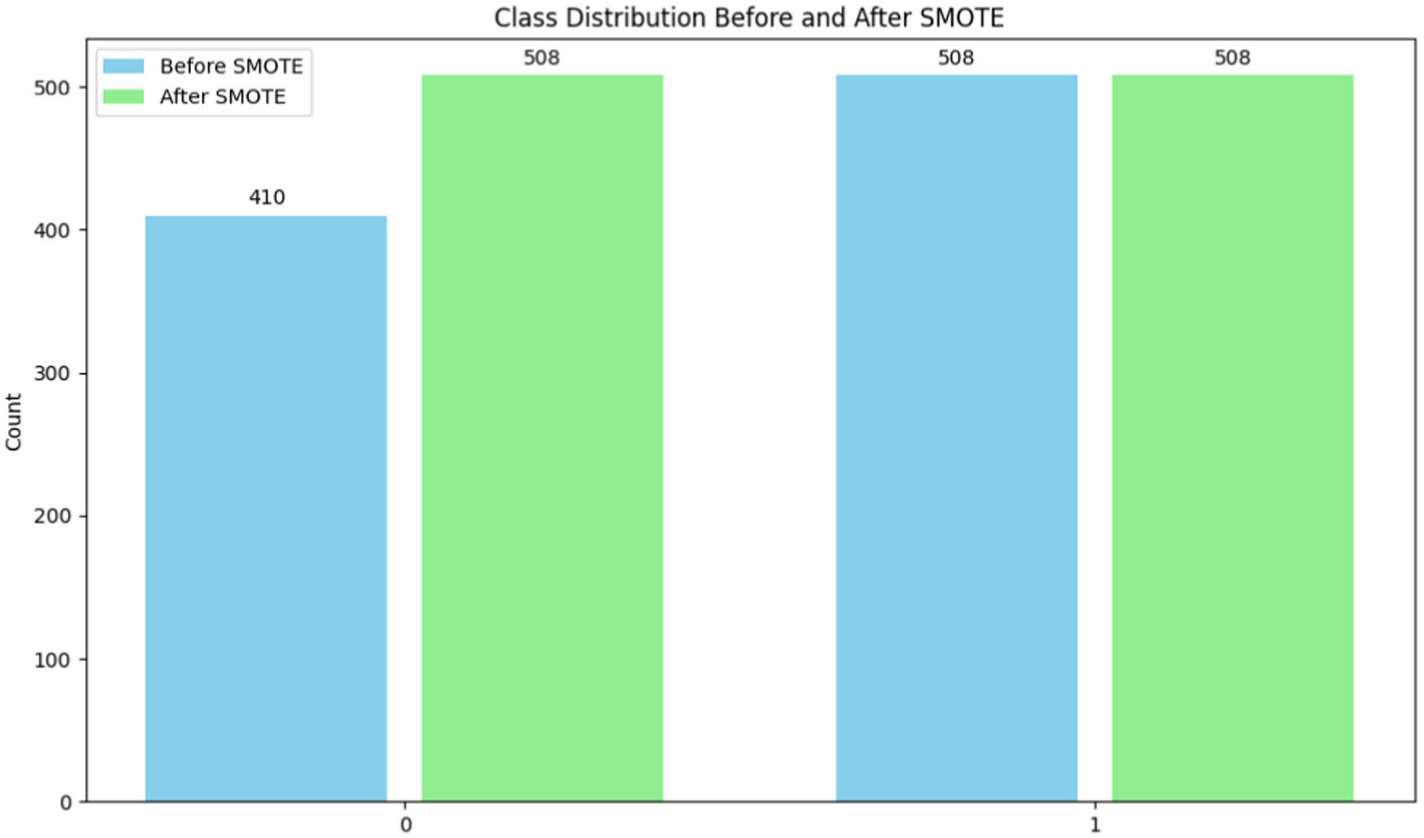
Before and after applying SMOTE.

To ensure that machine learning algorithms are not biased toward the dominant class and hence reduce prediction accuracy, imbalanced datasets must be handled. In order to rectify class imbalance, particularly in cases when minority occurrences are underrepresented, this system applies the Synthetic Minority Over-sampling Technique (SMOTE) (25) [shown in equation (6)]. Class distribution has an imbalance with 508 class 1 instances and 410 class 0 instances (shown in Table 2 and Figure 7). It would indicate that the 0.8071 imbalance ratio is less than the 1 - imbalance_threshold threshold. SMOTE manipulates the underrepresented class’s dataset presence by creating false instances of it. This is accomplished by building artificial instances along line segments that connect instances of minority classes. With a more evenly distributed dataset, the model may learn from more examples and, perhaps, make better predictions with new data.

**Table 2:** Before and after applying SMOTE.

Before applying SMOTE		After applying SMOTE	
Class	Count	Class	Count
0	410	0	508
1	508	1	508

Model prediction is improved with SMOTE (26) because it decreases class imbalance. When data from minority groups is limited, this strategy really shines in terms of model performance. To aid in the management of unbalanced datasets, SMOTE encourages correct and equitable predictions across all classes.


(6) $$\begin{array}{l} \mathrm{Imbalanced Ratio}\;\left(\mathrm{IR}\right)\\ =\frac{\mathrm{The count of occurrences in the majority class}}{\mathrm{The count of occurrences in the minority class}}\; \end{array}$$

#### 3.2.5 Feature selection using hybrid GOL2-2 T

A new hybrid feature selection approach called the Hybrid GOL2-2 T, in which L2 regularization is fused with the Grasshopper Optimization Algorithm (GOA) (27), is discussed. This solution of the metaheuristic attracts a promising subset of the feature set through the application of an objective function and global search. We then applied L2 regularization to the selected feature set. Majorly, the objective of L2 regularization is to penalize too many coefficients, promote sparsity, and preserve only the most useful features. Hybrid GOL-2 T combining fine tuning powers from L2 regularizations with the muscular strength of GOA combined gives a dependable feature selection technique. In this respect, models that provide predictive classification via two-level approaches should have higher classification accuracy and dependability since they help in selecting the most relevant characteristics and reducing overfitting. As has been correctly pointed out, for these reasons, this approach has gained significant acceptance and has become an indispensable tool for many machine learning applications, like regression and classification tasks.

#### 3.2.6 Grasshopper optimization algorithm

Developed in 2017 by Saremi et al. (32), the Grasshopper Optimization Technique (GOA) is a metaheuristic optimization technique inspired by nature. The idea originated from the way grasshoppers behaved in unison. GOA has been used to solve a variety of optimization problems, including feature selection in the context of machine learning. Here is a brief description of how GOA works shown in Algorithm 1, complete with formulas and the algorithm itself:

##### Algorithm 1: Grasshopper Optimization Algorithm (GOA)

Initialize population of grasshoppers (solutions)
  Initialize best solution (best_solution)
  Initialize number of iterations (iterations)
**While (termination criterion is not met)**
**    For each grasshopper ii**
          Calculate social interaction component *S_ii_* shown in equation (7)
          Calculate gravity component *G_ii_* shown in equation (8)
          Calculate wind component *A_ii_* shown in equation (9)
          Calculate movement of grasshopper ii (*x_ii_*)
          Update position of grasshopper ii (*x_ii_*)
          Evaluate objective function for new position (*fitness_ii_*)
          **If** ((*fitness_ii_*) > **fitness of best_solution)**
              Update best solution (best_solution)
          **End If**
      **End For**
      Update number of iterations (iterations)
**End While**
          Return best solution

(7) $$S_{ii}=C*\left(\frac{sum\left(x_{jj}-x_{ii}\right)}{N}\right)\;\;$$

(8) $$G_{ii}=-g*\left(x_{ii}-o\right)$$

(9) $$A_{ii}=U*\left(x_{e}-x_{ii}\right)$$

Where,

- **c** is a decreasing coefficient that balances the processes of exploration and exploitation.
- g is a constant that determines the strength of the gravity component is the center of the search space.
- U is a constant that determines the strength of the wind component.
-
$x_{e}$ is the position of the best solution found so far.
- N is the number of grasshoppers.
-
$x_{ii}\;$ and $x_{jj}$ are the positions of the grasshoppers.

The algorithm generates grasshoppers, each representing a possible solution. The first grasshopper in the population gets the best answer. The algorithm then loops through each grasshopper in the population. The application calculates grasshopper social interaction, gravity, and wind components. These components steer the grasshopper toward the best alternative.

**Figure 8:**
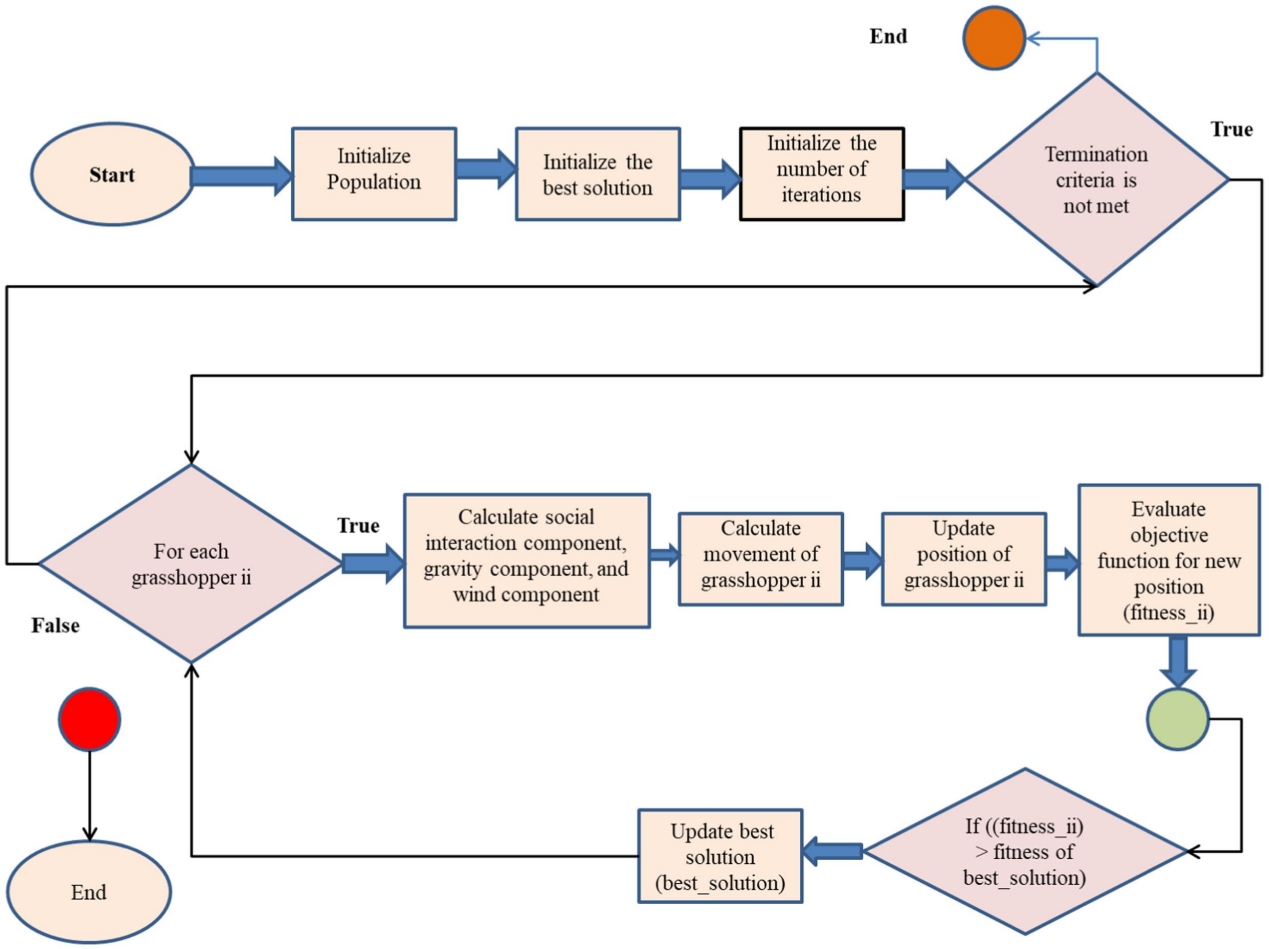
Feature selection flow chart for Grasshopper optimization algorithm.

The components calculated in the previous stage are used to modify the grasshopper’s movement. The objective function measures grasshopper positioning and solution efficacy. A new site becomes the ideal option if it outperforms the old one. After reaching grasshopper population termination criteria, the technique continues iteratively. A maximum number of iterations, a minimum fitness value, or any other suitable stopping condition may be used for the job. After optimization, the technique returns the ideal answer (Figure 8).

#### 3.2.7 L2 regularization

L2, sometimes called ridge regression (28), is a machine learning technique used to reduce a model’s complexity by adding a penalty term to the loss function. The penalty term is directly correlated with the square of the magnitudes of the coefficients, encouraging the model to have smaller coefficients and reducing the likelihood of overfitting shown in Algorithm 2.

The L2 regularization term is added to the loss function as shown in equation (10).


(10) Loss=MSE+(alpha∗sum(coefficient^2))

Where:

MSE is the mean squared error between the predicted and actual values shown in equation (11).

alpha is the regularization parameter (a hyperparameter).

Coefficient is the coefficient of the feature in the model.

The algorithm for L2 regularization can be described as follows:

##### Algorithm 2: L2 regularization

Initialize coefficients to small random values
**While (termination criterion is not met)**
      Calculate MSE using the current values of the coefficients by using equation (11)
      Calculate sum of squared coefficients by using equation (12)
      Calculate regularized loss function as the sum of the MSE and the regularization term (alpha * sum of squared coefficients)
      Update coefficients to minimize the regularized loss function
**End While**
  Return the optimized coefficients

(11) $$\mathrm{Mean Squared Error}\;\left(\mathrm{MSE}\right)=\frac{1}{n}\overset{n}{\underset{ii=1}{\sum }}{\left(y_{ii}-y_{ii}^{\iota }\right)}^{2}\;$$

(12) $$\mathrm{Sum}\;\mathrm{of squared coefficients}\;\left(\mathrm{SSC}\right)=\overset{p}{\underset{jj=1}{\sum }}\theta_{jj}^{2}\;$$

Where,

-
p is the number of coefficients.
-
$y_{ii}$ as the data point’s observed value *ii*
-
$y_{ii}^{\iota }$ as the anticipated value for data point *ii*.

**Figure 9:**
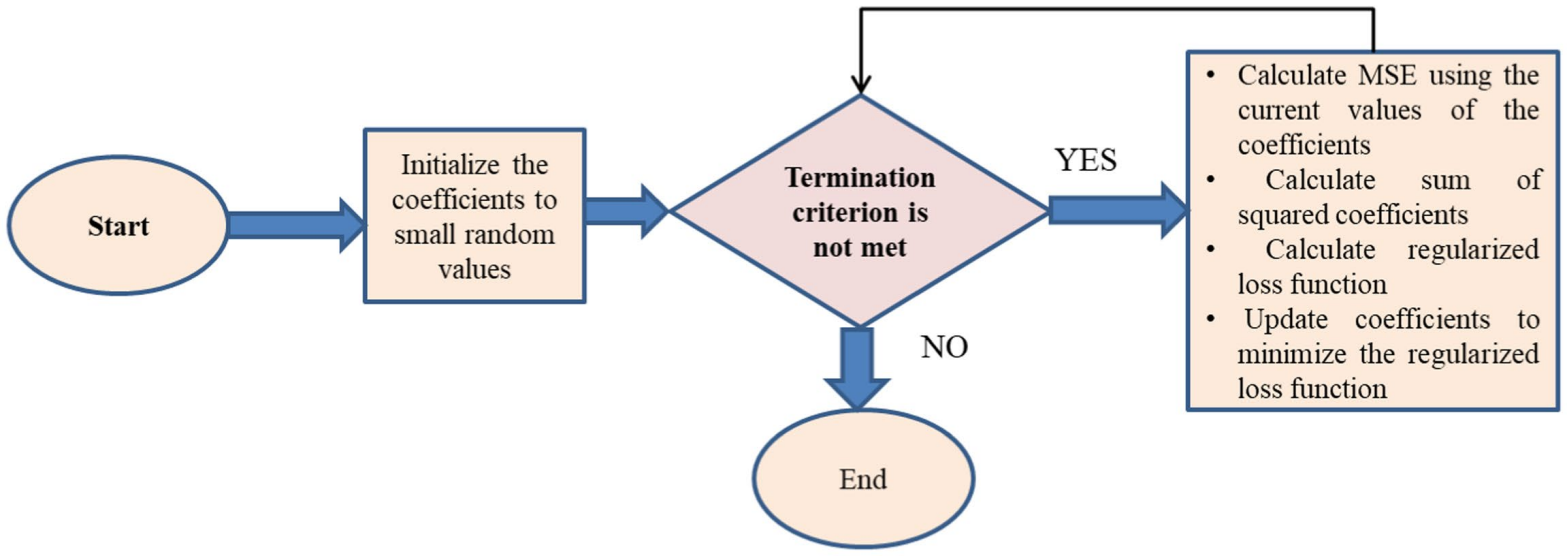
Feature selection flow chart for L2 regularization.

The L2 regularization approach may be used to a wide range of models due to its computational efficiency. To achieve the optimal balance between bias and variance, the regularization hyperparameter alpha has to be changed. Features that are more effective at lowering the Mean Squared Error (MSE) are chosen when L2 regularization reduces the size of the model’s coefficients. L2 regularization may be used as a feature selection method by selecting only those features in the model that have coefficients greater than zero (Figure 9).

### 3.3 Hyperparameter tuning using babysitting algorithm

The babysitting Algorithm (BA) (29) in AdaBoost (30) decision fusion manually evaluates the model’s performance after iteratively modifying the hyperparameters. Setting hyperparameters, constructing a table, separating the dataset into training, validation, and testing sets, and progressively experimenting with different combinations are the steps. For each combination, an AdaBoost classifier is trained on the training set and assessed on the validation set using a performance metric. The hyperparameter table is updated when the trial number, hyperparameters, and performance measure change. Select the hyperparameters with the best validation set outcomes after all trials. The training and validation sets are utilized to train a new AdaBoost classifier using the optimum hyperparameters. For an impartial evaluation, the finished model is tested on the testing set shown in Algorithm 3.

#### Algorithm 3: Hyperparameter Tuning Babysitting on AdaBoost Decision Fusion

// Initialize hyperparameters and performance metric
  InitializeHyperparameters()
  // Initialize the hyperparameter table
  InitializeHyperparameterTable()
  // Main loop for hyperparameter tuning
  **while (stopping criterion not met) do**    // Iterate through hyperparameter combinations
      **for each hyperparameter combination do**        // Train AdaBoost classifier with current hyperparameters
          model = TrainAdaBoostClassifier(current_hyperparameters)
          // Evaluate the model's performance on the validation set
          performance_metric = EvaluateModelPerformance(model, validation_set)
          // Update hyperparameter table with current hyperparameters and performance metric
          UpdateHyperparameterTable(current_hyperparameters, performance_metric)
      **end for**    // Select best hyperparameters based on the highest performance metric
      best_hyperparameters = SelectBestHyperparameters()
      // Train AdaBoost classifier with the best hyperparameters on the combined training and validation sets
      best_model = TrainAdaBoostClassifier(best_hyperparameters, combined_training_validation_set)
      // Evaluate the final model on the testing set
      final_performance_metric = EvaluateModelPerformance(best_model, testing_set)
      // Update stopping criterion based on convergence or maximum iterations
      UpdateStoppingCriterion()
**end while**

### 3.4 Model building for heart failure prediction

#### 3.4.1 Ensemble technique with adaptive boosted decision fusion

“Adaptive Boosted Decision Fusion (31) is an advanced ensemble learning algorithm that effectively combines the principles of Adaptive Boosting (AdaBoost) and Decision Fusion.” To prioritize instances that are harder to classify, this innovative approach has the algorithm adaptively changing the weights [shown in equation (13)] given to less effective learners. When combined with decision fusion, ABDF sequential training method for weak models allows for the efficient integration of results from many decision-makers [shown in equations (14–18)]. The ultimate result is a very accurate and reliable prediction model that is both adaptable and resilient. One way to make the ensemble better is via adaptive boosted decision fusion, which uses iterative refinement and smartly gives different learners different weights depending on how well they do. When it’s critical to combine multiple decision-making viewpoints to get superior predicted outcomes, this method shines.

**Input**:

Training dataset: $D=\left\{\left(ux_{1},uy_{1}\right)\left(ux_{2},uy_{2}\right),\ldots \ldots ,\left(ux_{n},uy_{n}\right)\right\}.$

Where $ux_{i}\;$ the feature is vector and $uy_{i}$ is the corresponding label.

**Number of weak learners:** UT

**Initialization**:


(13) $$1.\mathrm{Initialize instance weights}:uw_{i}=\frac{1\;}{n}for\;i=1,2,3\ldots \ldots .n\;$$

2. Initialize an empty ensemble of weak learners.


(14) For each iteration:ut=1,2,3…….UT:

3. Train a weak learner $uh_{t}$ using the current instance weights.


(15) $$\begin{array}{l} i.\;\mathrm{Compute the error of the weak learner}:\\ \in_{t}=\overset{n}{\underset{i=1}{\sum }}uw_{i}.|(uh_{t}\left(ux_{i}\right)\neq uy_{i} \end{array}$$

where ꟾ(.) is the indicator function.


(16) $$\mathrm{ii}.\;\mathrm{Compute the learner weight}:\alpha_{t}=\frac{1}{2\ln\left(\frac{1-\in_{t}}{\in_{t}}\right)}\;$$

(17) $$\begin{array}{l} \mathrm{iii}.\;\mathrm{Update instance weights}:\\ for\;i=1,2,3\ldots \ldots .\;n,uw_{i}\leftarrow uw_{i}.\exp\left(-\alpha_{t}.uy_{i.}\;uh_{t}\left(ux_{i}\right)\right)\; \end{array}$$

(18) $$\mathrm{Normalize weights}:uw_{i}\leftarrow \frac{uw_{i}}{\sum_{i=1}^{n}uw_{i}}\;$$

iv Add the weak learner $uh_{t}$ to the ensemble with weight $\;\alpha_{t}$ .

**Output:**


(19) $$\mathrm{Ensemble of weak learners}:\left\{\left(\alpha_{1},uh_{1}\right)\left(\alpha_{2},uh_{2}\right),\ldots \ldots ,\left(\alpha_{T},uh_{UT}\right)\right\}\;$$

**Predictions:**


(20) $$\begin{array}{l} \;\mathrm{For}\;a\;\mathrm{new}\;\mathrm{instance}\;ux,\mathrm{the final prediction is given}\;\mathrm{by}:\\ H\left(ux\right)=\sin(\overset{UT}{\underset{ut=1}{\sum }}\alpha_{t}\left(ux\right) \end{array}$$

This method combines the best features of AdaBoost and Decision Fusion in a way that strengthens the ensemble (26), making it better at handling misclassifications and making accurate predictions. A long-lasting ensemble model that frequently outperforms individual models is produced by ABDF iterative method of correcting errors of weak models [shown in equation (19)]. Classification problems, such as the prediction [shown in equation (20)] of cardiac illness, frequently use ABDF. It finds usage in a variety of domains due to its flexibility in accommodating varied poor learners (Tables 3, 4).

**Table 3:** Selected features with scores using GOA.

Features	Score
cp	0.047363
trestbps	0.002171
chol	0.000873
thalach	0.007371
oldpeak	0.047905
ca	0.03526

**Table 4:** Selected Features with Scores using L2 regularization.

Feature	Score
cp	0.051832
oldpeak	0.047056
ca	0.037252
thalach	0.007355
trestbps	0.002556

## 4 Result and discussion

### 4.1 Performance assessments

#### 4.1.1 Feature selection outcome using GOL2-2 T

**Figure 10:**
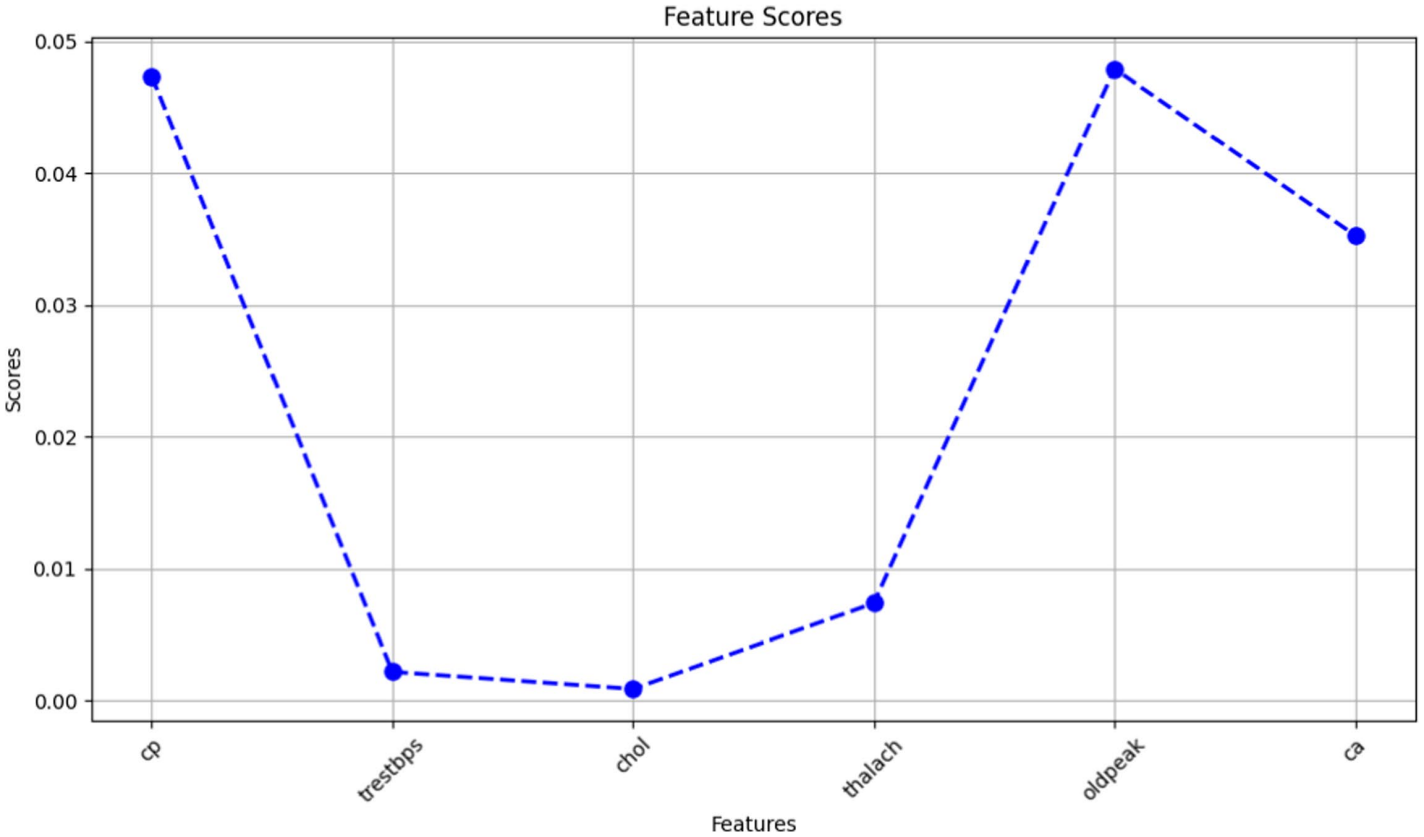
A line graph denoting selected features with scores using GOA.

**Figure 11:**
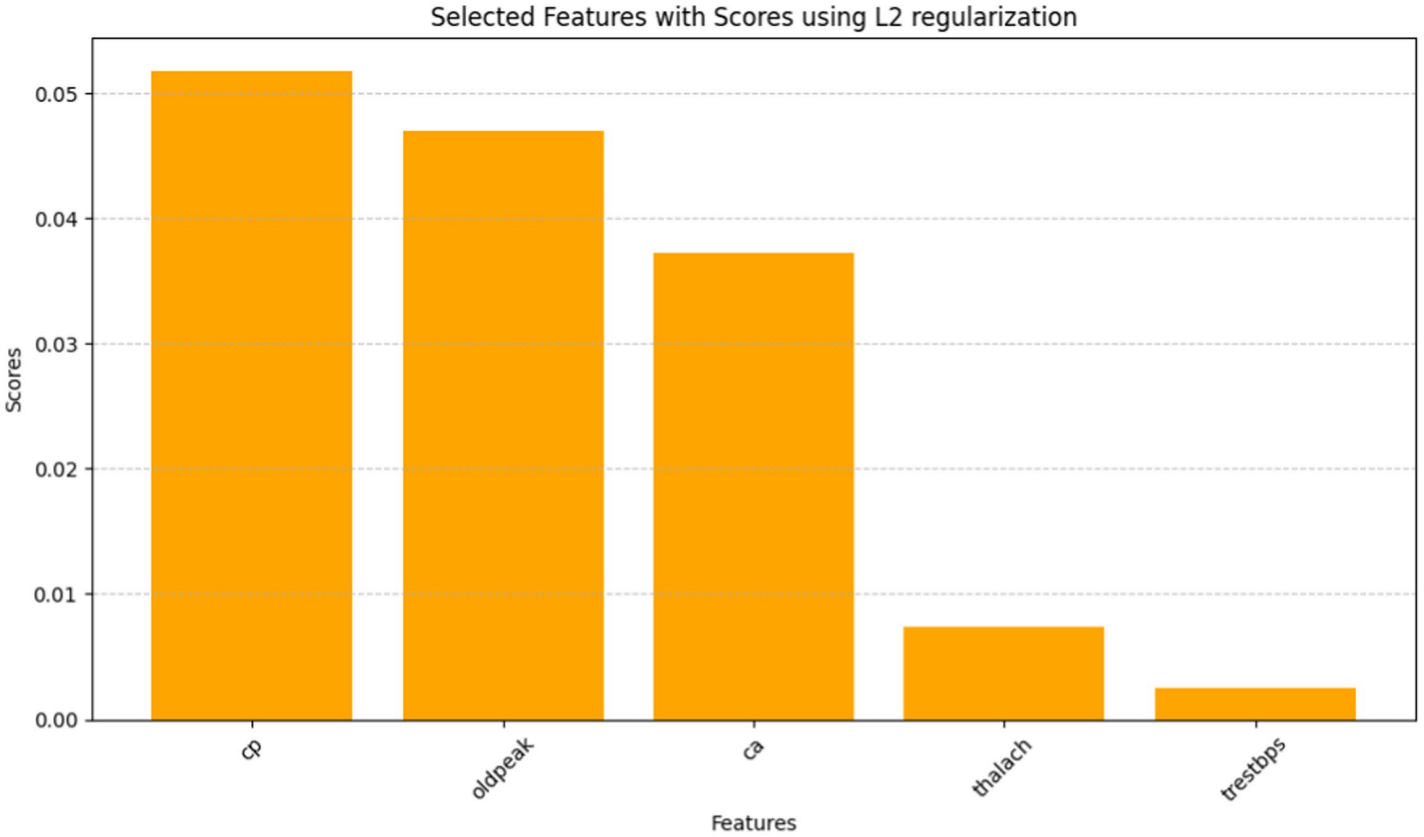
A bar graph denoting selected features with scores using L2 regularization.

The Grasshopper Optimization Algorithm (GOA) (32) identified heart disease predictors. This method found critical characteristics like chest pain type (cp), resting blood pressure (trestbps), serum cholesterol (chol), maximum heart rate (thalach), ST depression caused by exercise compared to rest (oldpeak), and the number of main vessels colored by fluoroscopy (ca). High scores showed relevancy. The prediction model ranked attributes by score. Next, we used ridge regression, also known as L2 regularization, to enhance feature selection. Revised features included oldpeak, thalach, ca, trestbps, and cp. Revaluating characteristics using L2 regularization yielded scores that accurately represent their value in heart disease prediction. Comparing the two feature selection approaches shows convergence in the selected qualities, suggesting they may be essential for heart disease identification. However, slight discrepancies in feature significance showed that GOA and L2 regularization use different techniques and criteria. We need more study to evaluate the predictive modeling of the upgraded features and the implications for heart disease diagnostics (Figures 10, 11).

#### 4.1.2 Hyperparameter tuning outcome using babysitting algorithm on ABDF

**Figure 12:**
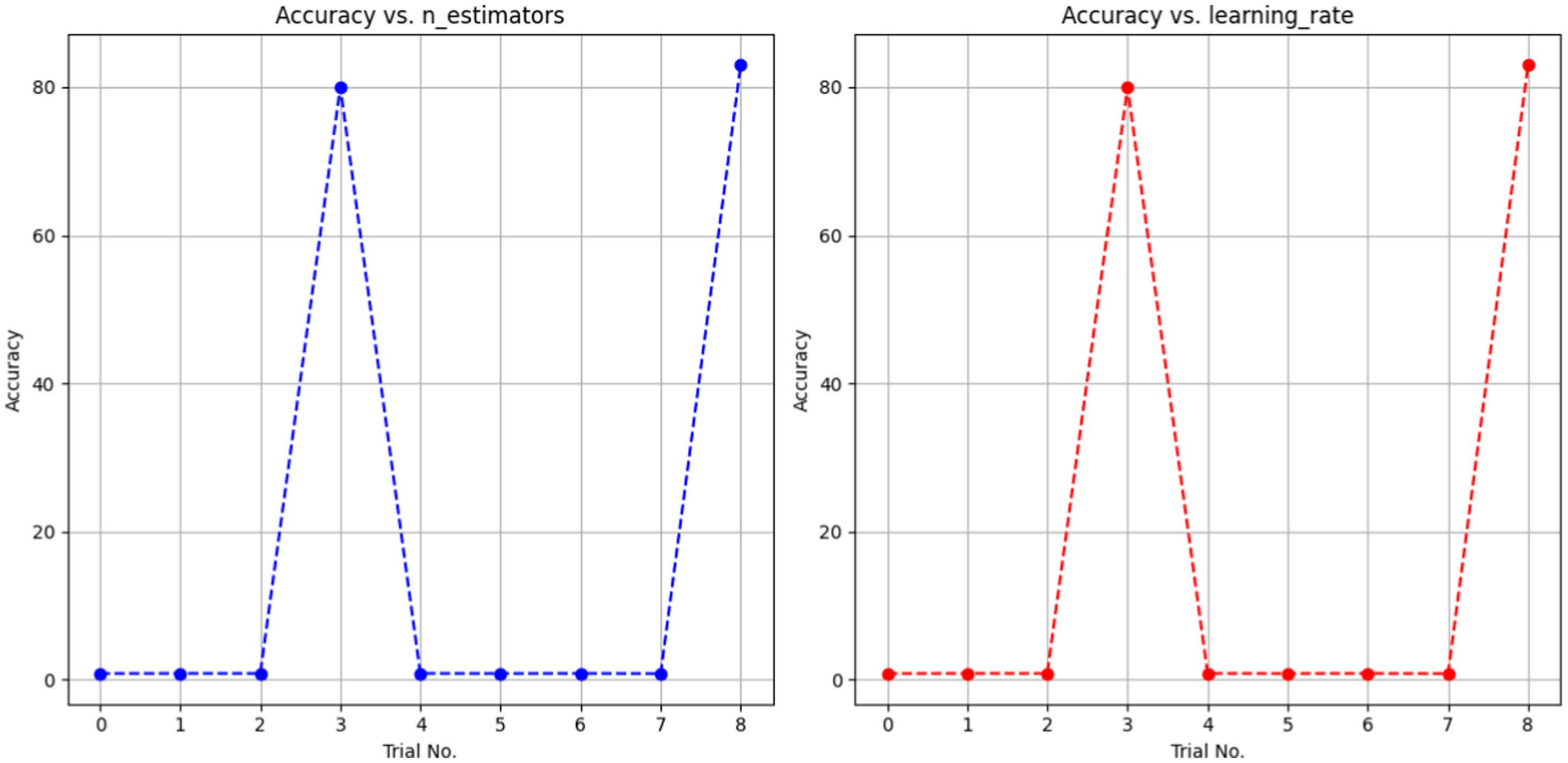
A dotted line graph denoting ABDF hyperparameters with babysitting.

The AdaBoost Decision Fusion model’s hyperparameters were optimized by a two-pronged approach involving tuning the n_estimators and learning rate with the help of the Babysitting Algorithm (see in Table 5). A narrow range of the search space for n_estimators, which was from 50 to 200, and a more broad range of the learning rate, which was from 0.5 to 1, was seen. The hyperparameter optimization was made through a number of runs by substituting various combinations of parameters for n_estimators and learning_rate (see in Table 6 and Figure 12). The data obtained from the ABDF model showed deviation across the many attempts conducted in the experiment; Trial No. 8 gave 8 as the most accurate results, their accuracy being 83.00%. The crucial aspiration of this process was the attainment of an optimal accuracy and robustness model for the ABDF model, specifically as it concerned the given task.

**Table 5:** AdaBoost decision fusion model hyperparameters tuning summary.

Models used	Hyperparameters tuning algorithm	Hyperparameters	Search Space
AdaBoost decision Fusion	Babysitting	n_estimators	50–200
		learning_rate	0.5–1

**Table 6:** AdaBoost decision fusion hyperparameters with babysitting.

Trial no.	Accuracy	n_estimators	learning_rate
0	0.802	50	0.1
1	0.82	50	0.5
2	0.812	50	1
3	80.00	100	0.1
4	0.822	100	0.5
5	0.804	100	1
6	0.819	200	0.1
7	0.79	200	0.5
8	83.00	200	1

### 4.2 IQR outlier detection with ABDF

**Figure 13:**
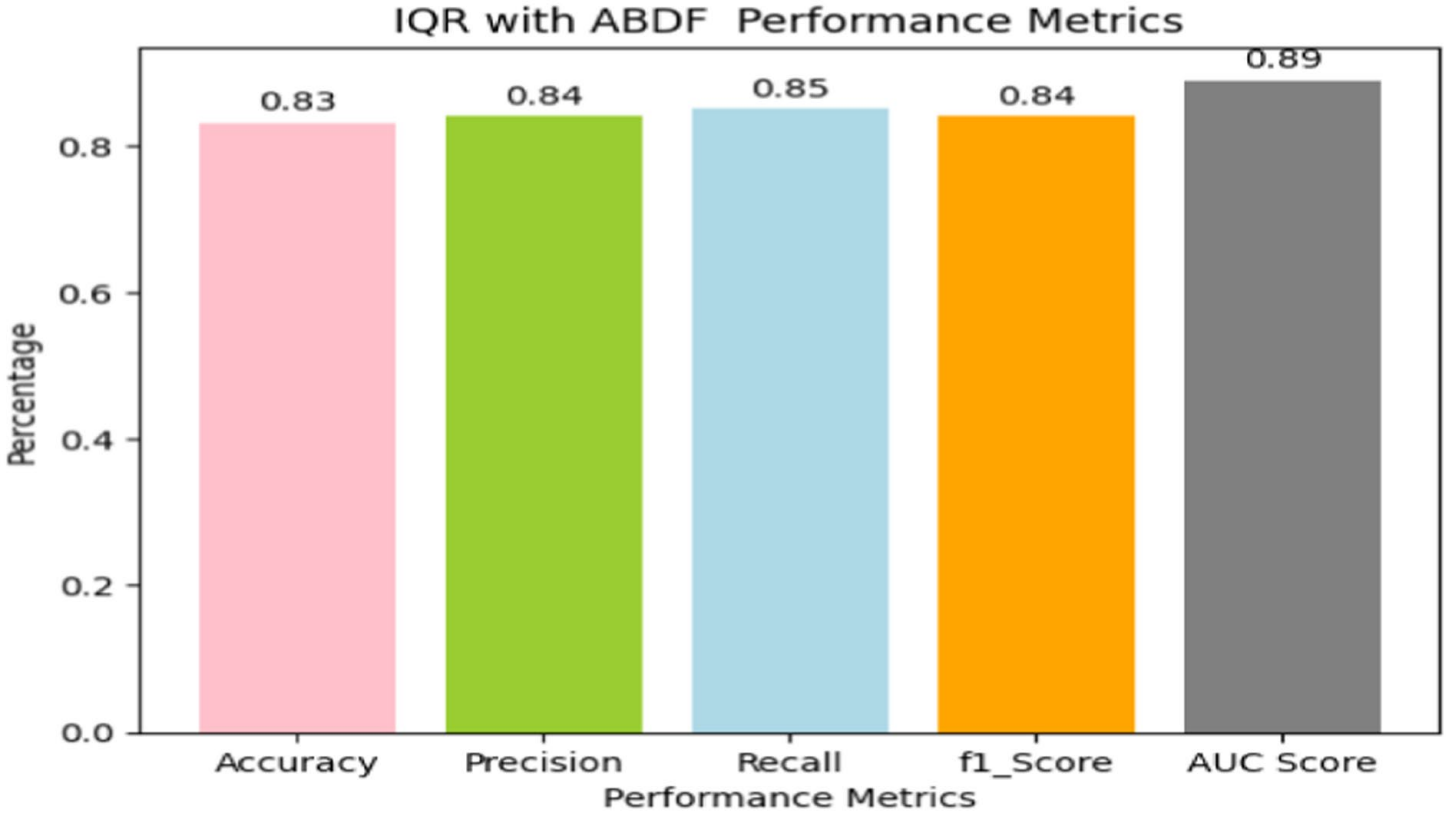
Bar graph shows IQR outlier detection with ABDF performance metrics.

**Figure 14:**
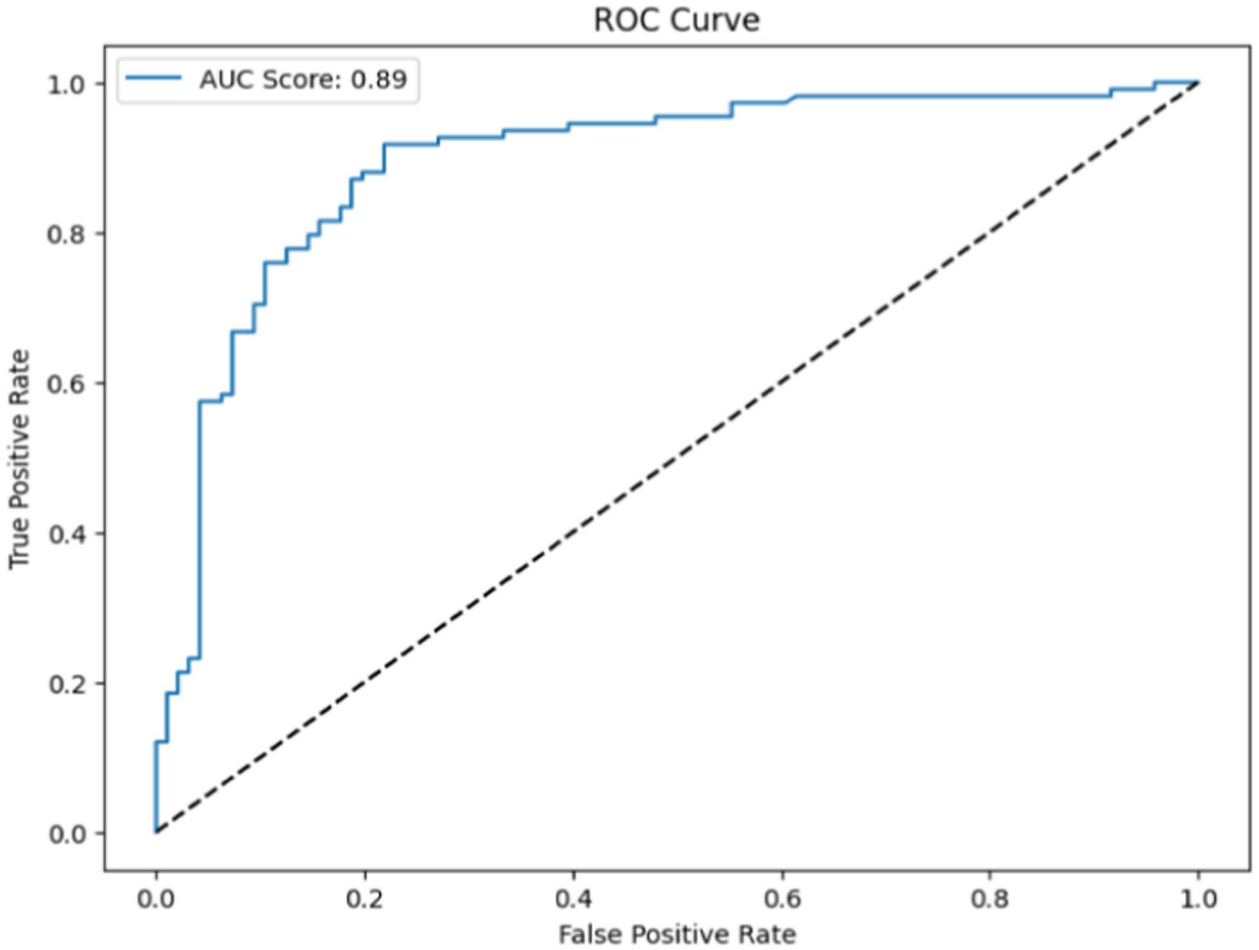
ROC for IQR outlier detection with ABDF.

Heart disease may be reliably predicted using the ABDF method and the IQR outlier preprocessing strategy. The model achieves an 83% accuracy rate in instance categorization and an 84% success rate in accurately anticipating predicted positives (see in Table 7 and Figure 13). The model correctly identifies a large number of positive examples, as evidenced by its impressive recall score of 85%. An F1 Score of 84% (a measure of both recall and accuracy) indicates that the model is performing well. With an Area Under the Curve (AUC) score of 89% (see in Figure 14), the model clearly can differentiate between positive and negative occurrences. Based on these metrics, it appears that preprocessing using ABDF and IQR improves the accuracy, precision, recall, and overall predictive performance of models used to forecast cardiac diseases. According to its reliable performance, the model may be relied on by healthcare providers to aid in the rapid identification and treatment for people at risk of heart disease.

**Table 7:** IQR outlier detection ABDF performance metrics.

IQR outlier detection with ABDF results	
Metrics	Values
Accuracy	0.83
Precision	0.84
Recall	0.85
f1_score	0.84
AUC Score	0.89

#### 4.2.1 Comparison of proposed method and other methods on heart disease dataset

In Table 8, multiple approaches are used to a heart disease dataset to assess accuracy, precision, recall, and F1-score. The suggested technique outperforms the others with 83.0% accuracy. This shows that it locates dataset instances properly. This method outperforms the Classification Tree and Artificial Neural Network (ANN) methods in classification testing. The new approach outperforms previous methods in accuracy, recall, and F1-score. Its great overall performance is due to its balanced trade-off between precisely recognizing positive examples (precision) and capturing all positive occurrences (recall).

**Table 8:** Comparison of proposed method and other methods on heart disease dataset.

Algorithm	Accuracy	Precision	Recall	f1_score
Classification tree (33)	77.0	79.0	79.0	79.0
ANN (17)	77.39	78.30	77.40	76.90
NB (34)	81.25	57.89	73.33	32.35
Proposed method	83.0	84.0	85.0	84.0

**Figure 15:**
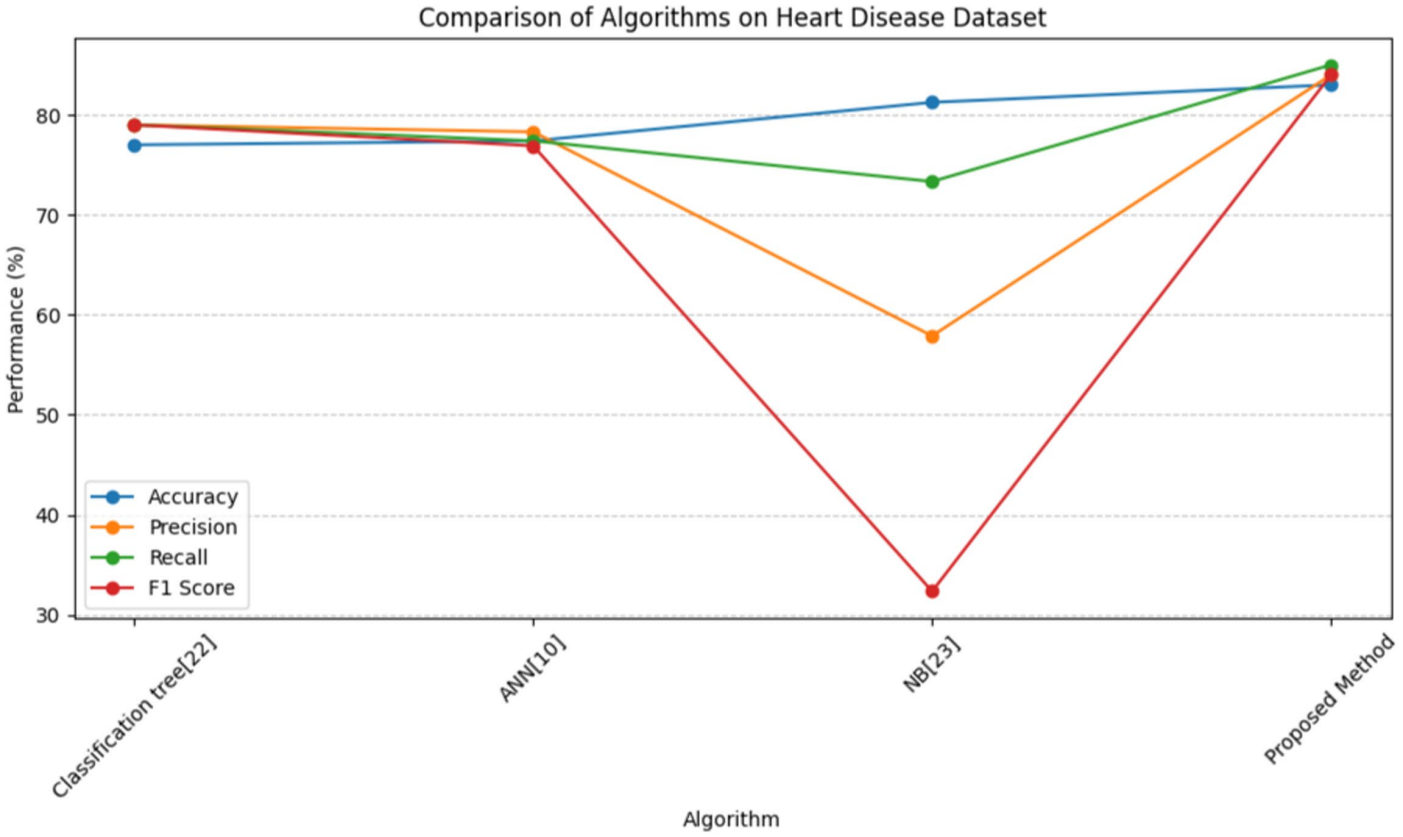
Line graph for comparison of proposed method and other methods on heart disease dataset.

**Figure 16:**
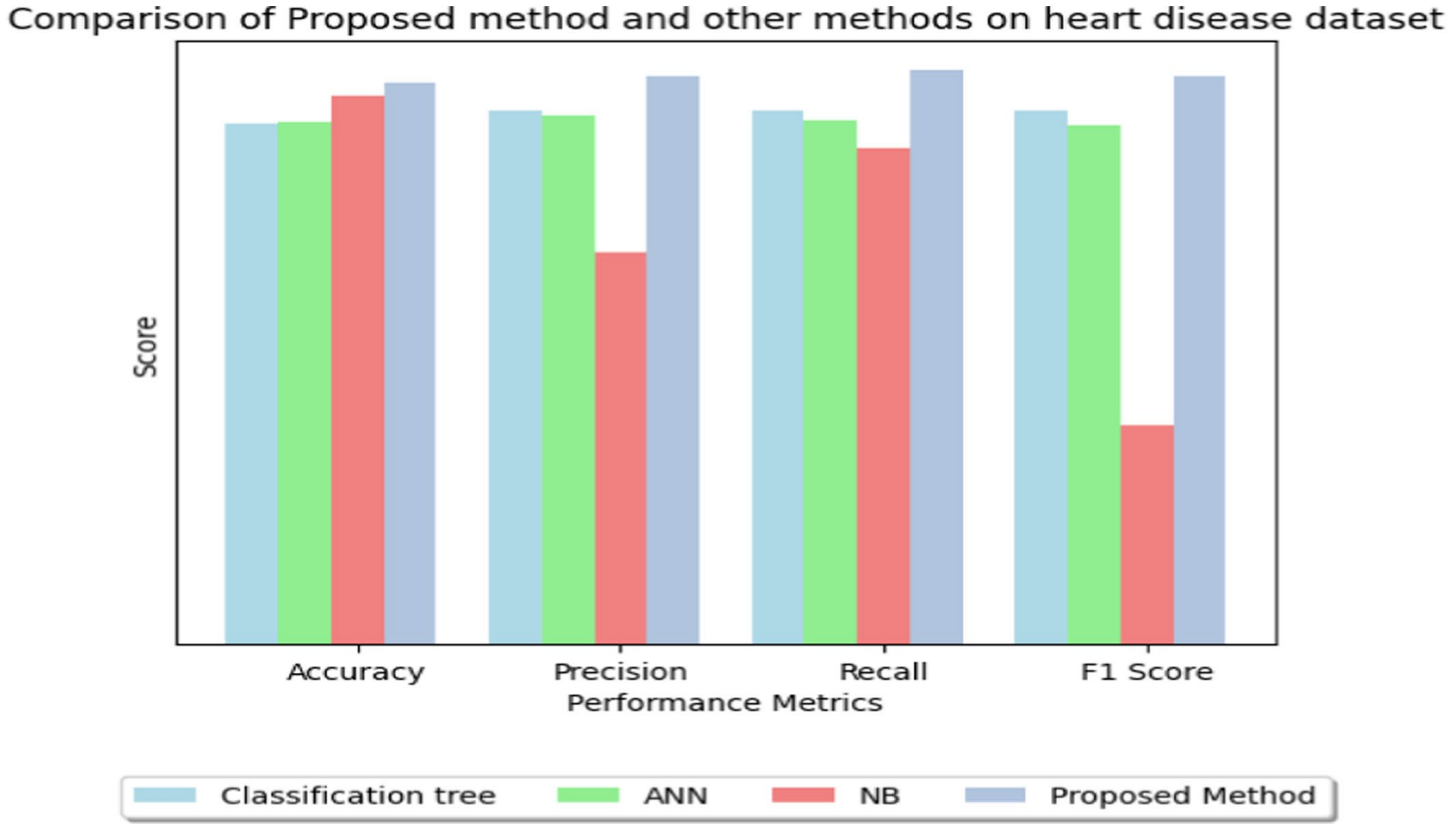
Bar graph for comparison of proposed method and other methods on heart disease dataset.

The Naive Bayes (NB) technique exceeds the suggested method in accuracy (81.25%) but much worse in precision, recall, and F1-score. More particular, the NB technique has poorer precision and F1-score than the suggested strategy, suggesting more false positives and a worse accuracy-recall trade-off. The findings suggest that the proposed technique balances accuracy and precision-recall, making it suitable for heart illness classification (see in Figures 15, 16). The comparison research also emphasizes the need of choosing the right technique for favorable performance indicators. This scenario shows that the recommended strategy is better than the present options.

## 5 Discussions

Our work presents an 83% reliable machine learning heart disease prediction approach. We used cutting-edge methods like SMOTE, IQR outlier detection, MICE, and GOL2-2 T, a hybrid feature selection technique, to improve predictive accuracy and robustness. Combining these techniques improved feature selection and model performance, according to our findings. Our heart disease patient identification approach is very accurate. These results demonstrate the need of using cutting-edge machine learning algorithms in medicine to identify and cure diseases early.

Our findings may help doctors predict cardiac disease, improving patient care and intervention. Our accurate diagnostic equipment may enhance patient outcomes and minimize cardiovascular disease mortality. However, our research has some drawbacks. Our hopeful results are limited to a dataset and may not apply to other patient populations or healthcare situations. Data quality and feature selection criteria may also affect our model’s performance.

We urge additional research to corroborate our results across a variety of datasets and populations. Using additional machine learning methods (35–40) and domain-specific information may improve the model’s interpretability and prediction accuracy. To evaluate the long-term effects of early cardiac disease identification on patient outcomes, longitudinal studies are needed. In conclusion, our results emphasize the necessity for ongoing study to develop cardiovascular prediction analytics.

## 6 Conclusion and future scope

In conclusion, our study met the urgent demand for precise and effective cardiovascular disease prognostic diagnostic tools. MICE, IQR outlier detection, SMOTE, and Adaptive Boosted Decision Fusion (ABDF) were used to improve heart disease prediction models’ precision and reliability. The Hybrid GOL2-2 T feature selection technique has enhanced our process by discovering important features and decreasing overfitting.

We solved class imbalance, missing data, and outlier identification to create a model that outperforms previous methods. The accuracy rate of 83.0% and balanced F1 score of 84.0% of our heart disease prediction method were impressive. The accuracy, recall, and AUC score demonstrate the validity and applicability of our methods. Our findings show that powerful machine learning techniques must be used in healthcare to produce reliable cardiovascular disease diagnosis tools. The study gives doctors tools for early diagnosis and effective treatment of cardiovascular disease risk.

Future study may improve prediction models and examine additional factors to improve diagnostic precision.

## Data availability statement

The original contributions presented in the study are included in the article/supplementary material, further inquiries can be directed to the corresponding authors.

## Author contributions

SP: Visualization, Data curation, Investigation, Software, Validation, Writing – original draft. MH: Data curation, Investigation, Funding acquisition, Project administration, Supervision, Writing – review & editing. SA: Writing – review & editing. US: Conceptualization, Software, Validation, Writing – original draft. NT: Conceptualization, Formal analysis, Investigation, Writing – review & editing. SI: Investigation, Methodology, Resources, Visualization, Writing – review & editing. FA: Resources, Software, Validation, Writing – review & editing. TA: Conceptualization, Data curation, Formal analysis, Writing – review & editing. AN: Data curation, Investigation, Writing – review & editing. GS: Writing – review & editing. CY: Funding acquisition, Writing – review & editing, Conceptualization, Formal analysis, Resources, Visualization. TG: Formal analysis, Methodology, Validation, Writing – review & editing.
